# One tube for all: 1‐year outcomes after transition to Paul glaucoma implant at a tertiary centre

**DOI:** 10.1111/aos.17443

**Published:** 2025-01-24

**Authors:** Anne Studsgaard, Stine Elkjær Nielsen, Niklas Telinius

**Affiliations:** ^1^ Department of Ophthalmology Aarhus University Hospital Aarhus Denmark

**Keywords:** glaucoma drainage device, glaucoma surgery, PAUL® glaucoma implant, primary open‐angle glaucoma, secondary glaucoma

## Abstract

**Purpose:**

To evaluate the intraocular pressure (IOP) lowering effect and success rate of Paul glaucoma implant (PGI) in refractory glaucoma after changing practice pattern from Ahmed and Baerveldt tubes to PGI.

**Methods:**

A prospective observational study of the first 50 consecutive PGI surgeries at a single Danish tertiary centre from January 2022 to October 2023. Primary endpoints were IOP and success rates after 12 months. Secondary endpoints were the use of IOP‐lowering medications and complications. All cases had a risk of failure for traditional glaucoma surgery (neovascular glaucoma, oil‐filled eye or uveitis).

**Results:**

Preoperative IOP was 29.9 ± 8.6 mmHg and the mean number of topical IOP‐lowering medications used was 3.4 ± 0.76 with 14 cases of systemic acetazolamide. Twelve months after surgery IOP was reduced to 11.4 ± 3.1 mmHg and complete success rate with (a) IOP≤21 mmHg was achieved in 43%, (b) IOP≤18 mmHg in 43%, (c) IOP≤15 mmHg in 41% and (d) IOP≤12 mmHg in 33%. Qualified success rate (on topical glaucoma medications) was achieved in (a) 96%, (b) 94%, (c) 86% and (d) 71% of the cases.

The number of topical IOP‐lowering medications was 0.9 ± 0.9 after surgery and 47% were medication free. Early (<3 months) and late (>3 months) complications were observed in 22% and 16% of patients respectively.

**Conclusion:**

This study indicates that PGI provides a good IOP‐lowering effect after 12 months in a population with risk factors for failure for traditional glaucoma surgery.

## 1 INTRODUCTION

The use of glaucoma drainage devices (GDD) in patients with or without prior incisional surgery has increased over the past two decades based on the favourable outcomes of the TVT and PTVT studies (Gedde et al., 2012; Gedde et al., 2022). The non‐valved Baerveldt glaucoma implant (Johnson & Johnson, California, USA) and the valved Ahmed glaucoma implant (New World Medical, California, USA) were introduced in the early 1990s and are to date the most commonly used devices. In 2018 the non‐valved Paul glaucoma implant (PGI, Advanced Ophthalmic Innovations, Singapore, Republic of Singapore) attained CE Mark in Europe, but is still not FDA approved. The PGI has a smaller internal (ID = 0.127 mm) and external tube diameters (ED = 0.467 mm) than those of the Ahmed glaucoma implant and Baerveldt glaucoma implant (ID = 0.305 mm, OD = 0.635 mm). The endplate is similar to that of the Baerveldt in terms of surface area (342mm^2^) and height (0.95 mm) but made of flexible silicone and with less of the plate placed under the recti muscles and thus more surface area available for bleb formation. The theoretical advantages of the PGI lie in the plate design and the smaller tube. A flexible plate of a low thickness may result in a lower risk of plate exposure and diplopia. The smaller tube results in the tube occupying less space in the anterior chamber angle which facilitates placement without involving Schwalbe's line or cornea endothelial periphery, thus possibly decreasing endothelial cell loss over time. The risk of conjunctival erosions may also be lower when the tube diameter is smaller and protrudes less on the ocular surface.

Although not tested in a randomised clinical trial, the few studies that have been published on the outcomes of the PGI demonstrate intraocular pressure (IOP)‐lowering effect comparable to Baerveldt and at least as safe in terms of complications.

Aarhus University Hospital is a tertiary referral centre for glaucoma surgery. Ahmed and Baerveldt shunts were the GDD of choice until PGI was introduced. The purpose of this study was to evaluate 1‐year outcomes of the first 50 consecutive PGI surgeries in terms of efficacy and safety.

## 2 MATERIALS AND METHODS

A single centre prospective observational study of all glaucoma patients undergoing PGI implantation at the Department of Ophthalmology, Aarhus University Hospital, Denmark, from January 2022 to October 2023. All patients underwent standardised pre‐defined clinical courses and data were collected according to pre‐defined criteria.

The recommended description and the core outcome set for innovative glaucoma surgeries developed by the European Glaucoma Society (EGS) were followed, including the STROBE Statement Checklist (Abegao Pinto et al., 2023).

At referral, all patients had undergone a full ophthalmic examination. Best corrected visual acuity (BCVA) was performed using the Snellen chart. Slit lamp examination, indirect ophthalmoscopy, IOP measurement using Goldmann applanation tonometry (mmHg) and gonioscopy were performed by a glaucoma specialist. A specular microscope (CEM‐530, Nidek, Gamagori, Japan) was used to measure endothelial cell density (ECD, cells/mm^2^) and visual field testing was performed using Humphrey 30–2 SITA Standard (HFA II‐i‐750, Carl Zeiss Meditec, Jena, Germany).

The documented baseline characteristics included age, sex, ethnicity, type of glaucoma, visual field (mean deviation), medicated IOP, number of IOP‐lowering medications, lens status, number and type of previous ocular surgeries, BCVA and ECD.

Data for this study were collected at 1 day, 1 week and 1, 3, 6 and 12 months after surgery. IOP, medication use and complications were registered at each visit. BCVA and ECD were measured at referral and 12 months postoperatively. The window for 12‐month visits was 12 months ±1 month.

The primary endpoints were IOP and success rates. Secondary endpoints were the use of IOP‐lowering medications and complications. According to EGS recommendation four different IOP thresholds were used for success: (a) IOP ≤21 mmHg, (b) IOP ≤18 mmHg, (c) IOP ≤15 mmHg and (d) IOP ≤12 mmHg. Complete success was defined as achieved target IOP without additional IOP‐lowering medication, and qualified success with additional IOP‐lowering medication.

Complications were defined as general for glaucoma surgery or procedure specific for GDD, according to A Guide on Surgical Innovation for Glaucoma by EGS (Abegao Pinto et al., 2023). The general complications included choroidal detachment, anterior chamber (AC) shallowing, clinical hypotony (IOP <6 mmHg associated with choroidal detachment and/or with hypotony maculopathy and/or with AC depth reduction), BCVA loss (reduction of >2 Snellen lines), macular oedema, hyphaema, ECD loss (mean loss and >20%, >30% and > 40% reduction), corneal decompensation and IOP spike (elevation of 10 mmHg from preoperative IOP). GDD‐specific complications included leak, ptosis, diplopia, dellen, dysesthesia, iridodialysis, iris atrophy, device obstruction, device malposition, device migration, device exposure and stent exposure.

Complications were defined as early if it occurred within 3 months of the surgery, and late when it occurred after 3 months.

The study was registered and approved as a quality control study in accordance with the Danish legislature.

### 2.1 Patients

All patients who underwent PGI surgery at our clinic in the described period were included. No specific inclusion criteria were used. During the study period, only one patient received a different tube than PGI (Ahmed FP7), based on a patient request for standard treatment while in the early phase of implementing PGI at the department.

### 2.2 Surgical approach

Surgery was performed by two surgeons (SEN and NT). No combined surgeries (e.g. with cataract surgery) were performed. The surgery was performed in retrobulbar anaesthesia with Carbocain (20 mg mepivacainhydrochlorid/ml). General anaesthesia was used if necessary. The PGI was preferably placed in the superotemporal quadrant and in the anterior chamber, but when not possible the inferonasal quadrant and/or sulcus was used. After the formation of a fornix‐based conjunctival peritomy, Mitomycin C (0.4 mg/mL) on an eye fluid wick (Visitec, 7.5 cm x 0.4 cm) was applied subconjunctival for 3 min. A Prolene 6–0 suture (Ethicon, 8711H) was inserted into the tube and the loose end was placed in the fornix inferior under the conjunctiva. The plate was placed under the superior and temporal recti muscles. VISCOAT (Alcon Laboratories, Texas, USA) was injected in the AC or Healon GV Pro (Johnson & Johnson, California, USA) behind the iris in cases with placement in the sulcus. With a 25G cannula a scleral tract was fashioned to the AC or via the sulcus to the posterior chamber. Ethilon 8–0 (Ethicon, B7714) sutures were used to fixate the plate and Ethilon 9–0 (Ethicon, 1717G) to fixate the tube to the sclera. The tube was covered with a 350 μm thick corneal lamellar graft, which was provided by the Danish Cornea Bank at Aarhus University Hospital. The conjunctiva was readapted with Vicryl 9–0 (Ethicon, V439G). Cefuroxime (1 mg) was administered intracamerally, and a combination of dexamethasone and hexamycine was given subconjunctivally at the end of the surgery.

### 2.3 Post‐operative course

Topical preservative‐free antibiotics (chloramphenicol) were used four times daily for 7–10 days. Topical preservative‐free steroids (dexamethasone) were used eight times daily for the first week, followed by 2 weeks of six times daily and then reduced to four times daily for 3 months after which the steroids were tapered out completely over 2 weeks.

Removal of the intraluminal stent was considered after 3 months. Prior to this IOP was strived to be under 14 mmHg using glaucoma medications, in the following order, when necessary: betablocker, carboanhydrase inhibitor, prostaglandine and brimonidine. After stent removal target pressure was aimed at <12 mmHg in cases with advanced glaucoma and < 17 mmHg in all other cases. Glaucoma medications were introduced as necessary to achieve this.

### 2.4 Statistical analysis

The statistical analyses were performed using GraphPad Prism 10.0. All data are presented as mean ± standard deviation unless stated otherwise. IOP and medication use were analysed with a one‐way ANOVA and Sidak's multiple comparison test. Subgroup analysis of IOP and medication use were analysed with a two‐way ANOVA with Sidak's multiple comparison test.

## 3 RESULTS

A total of 50 consecutive PGI surgeries from January 2022 to October 2023 were included in this study (*n* = 49). Two cases were lost to 1‐year follow‐up; one patient died and one patient had open globe trauma with loss of light perception. Preoperative IOP was 29.9 ± 8.6 mmHg, mean number of topical IOP‐lowering medications was 3.4 ± 0.8 and 14 (29%) cases were treated with systemic acetazolamide. Further baseline characteristics are shown in Table 1.

**TABLE 1:** Baseline characteristics of the study group.

Number of patients, *n*	49
Age (years)	
Mean ± SD	66.5 ± 16.9
Range	13–85
Sex, *n* (%)	
Male	38 (78)
Female	11 (22)
Ethnicity, *n* (%)	
Caucasian	44 (90)
Middle Eastern	4 (8)
South Asian	1 (2)
Glaucoma type, *n* (%)	
Primary open angle glaucoma	15 (30)
Primary angle closure glaucoma	1 (2)
Secondary glaucoma	33 (67)
Oil‐filled eye	13 (27)
Uveitic glaucoma	4 (8)
Neovascular glaucoma	8 (16)
Pseudoexfoliation glaucoma	2 (4)
After penetrating keratoplasty	2 (4)
Congenital cataract	1 (2)
Syndromes	3 (6)
Visual field, MD, dB	
Mean MD ± SD	−15.8 ± 6.1
Range	−3 to −27
Medicated IOP, mmHg	
Mean ± SD	29.9 ± 8.6
Range	18–50
IOP‐lowering medication	
Mean number of topical IOP‐lowering agents ± SD	3.4 ± 0.76
Acetazolamide, number of patients (%)	14 (29)
Lens status, *n* (%)	
Phakic	6 (12)
Pseudophakic	41 (84)
Aphakic	2 (4)
Previous glaucoma surgery, *n*, (%)	
No	26 (53)
Yes	23 (47)
Trabeculectomy	21 (43)
Trabeculotomy	1 (2)
Other GDD (Ahmed, Baerveldt)	2 (4)
Transscleral cyclophotokoagulation	1 (2)
BCVA, logMAR	
Mean ± SD	0.50 ± 0.45
Range	−0.1 to 2
ECD, cells/mm^2^	
Mean ± SD	2244 ± 416

Out of 50 PGI implantations, 42 PGIs were placed in the AC (39 in the superotemporal quadrant and 3 in the inferonasal quadrant), seven in sulcus and one in pars plana. One case had a second PGI implanted within 4 months due to insufficient pressure reduction.

### 3.1 Primary endpoints

**FIGURE 1:**
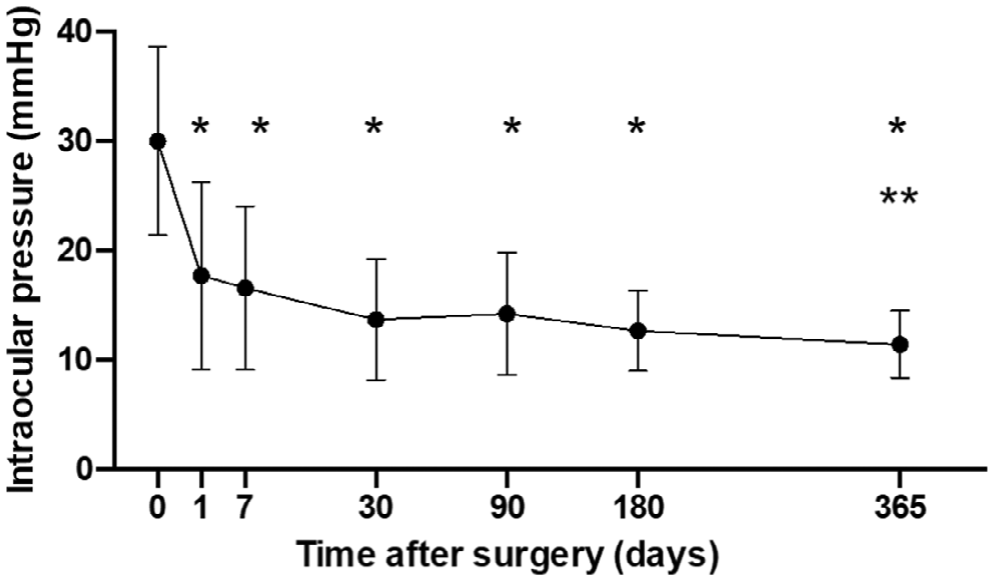
Mean intraocular pressure at each study time point. Error bars represent standard deviation. *p < 0.0001 compared with baseline. **p = 0.0413 compared with 3 months.

**FIGURE 2:**
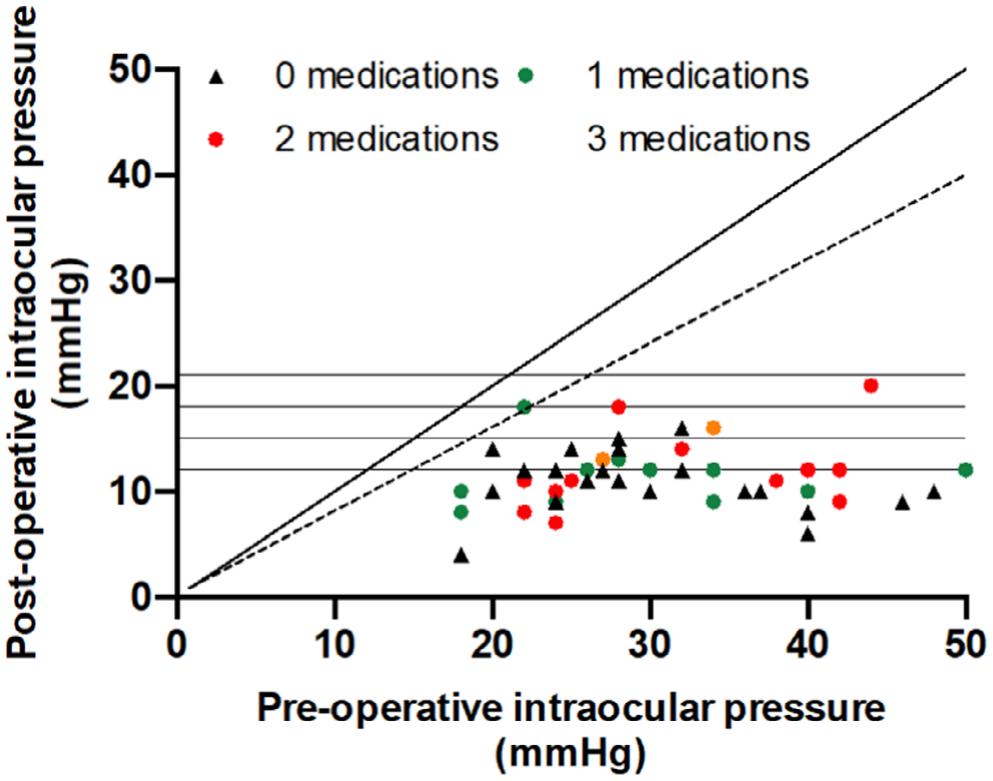
Scatter plot of 1‐year intraocular pressure plotted against pre‐operative intraocular pressure. Horizontal lines represent four intraocular pressure thresholds (≤21, ≤18, ≤15 and ≤ 12). Stippled line represents 20% intraocular pressure reduction. Colour represents the use of topical glaucoma medications.

IOP was significantly (*p* < 0.01) reduced at all timepoints from day one until the final follow‐up visit at 12 months (Figure 1). IOP at 12 months was 11.4 ± 3.1 mmHg. Mean IOP reduction at 12 months was 18.7 ± 8.9 (4–38) mmHg equivalent to a 59.6 (18–80)% reduction, see Table 2 for more details for the whole study period. Scatter plot of post‐operative versus pre‐operative IOP is presented in Figure 2. There was a significant IOP reduction (*p* = 0.0413) between the 3‐month visit and 12‐month visit consistent with a pressure drop after intraluminal Prolene stent removal. The intraluminal Prolene stent was removed in 42/49 cases at a median of 105 (interquartile range 95–120) days after surgery.

**TABLE 2:** Intraocular pressure and intraocular pressure reduction (%) at all study visits.

	Pre‐operative	Day 1	Week 1	Month 1	Month 3	Month 6	Month 12
IOP							
mmHg ± SD	29.9 ± 8.6	17.7 ± 8.5	16.6 ± 7.5	13.7 ± 5.5	14.2 ± 5.6	12.6 ± 3.7	11.4 ± 3.1
Range, mmHg	18–50	3–46	7–42	2–34	4–35	4–22	4–20
Reduction, %		39.4	41.5	51.9	49.2	54.8	59.6

**FIGURE 3:**
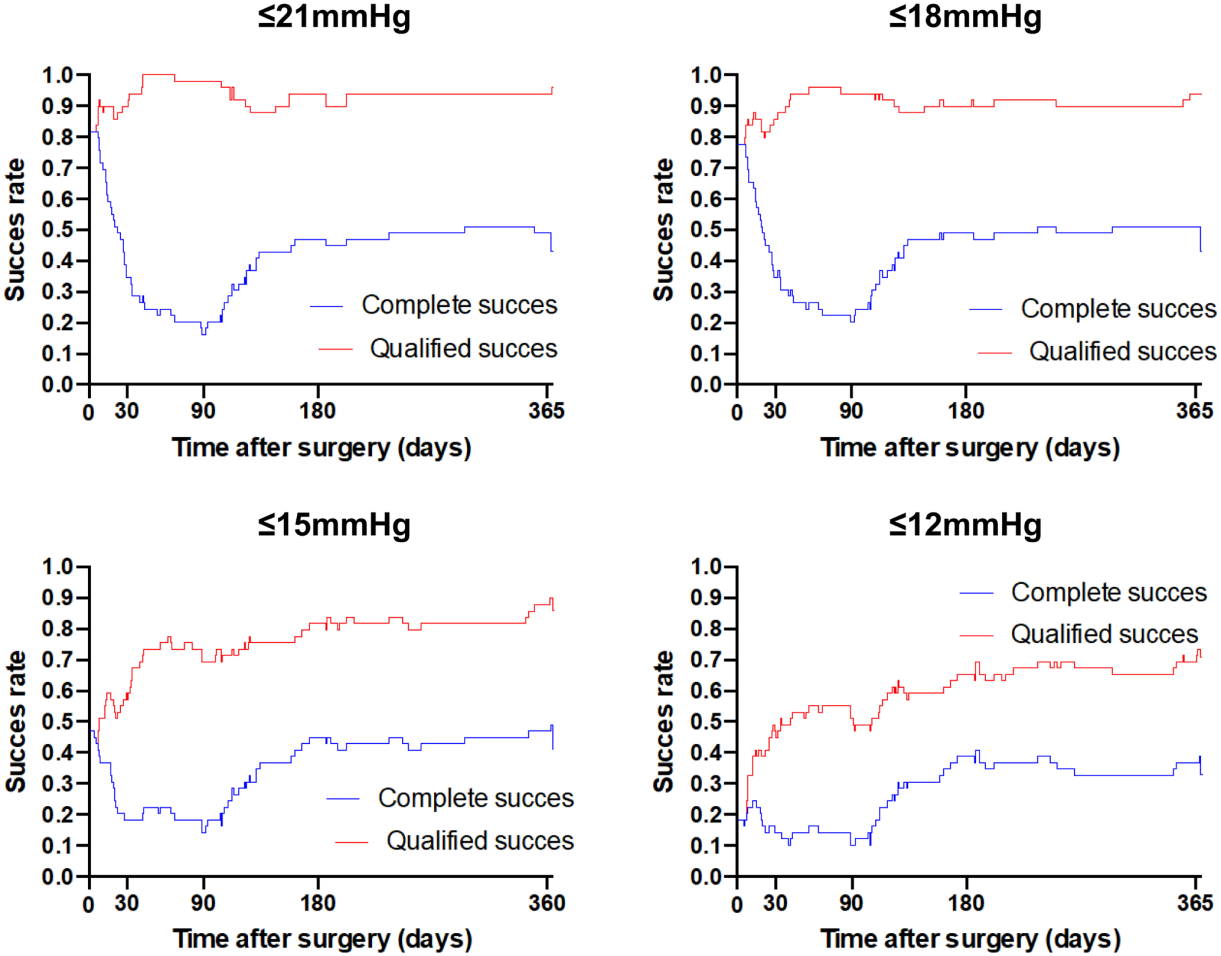
Kaplan–Meier curves for complete (blue) and qualified (red) success for different intraocular pressure criteria, with complete defined as no use of glaucoma medications and qualified with glaucoma medications. Complete success rates are low in the early period since the aim was to keep a pressure below 14 mmHg in the early post‐operative period, which for many patients required the use of glaucoma medications. The intraluminal Prolene 6–0 stent was removed in most patients after 3 months and the subsequent pressure drop lead to a reduced need for glaucoma medications and higher complete success rates.

At 12 months, complete success rate with (a) IOP≤21 mmHg was achieved in 43%, (b) IOP≤18 mmHg in 43%, (c) IOP≤15 mmHg in 41% and (d) IOP≤12 mmHg in 33%. Qualified success rate at 12 months was achieved in (a) 96%, (b) 94%, (c) 86% and (d) 71% of the cases. Kaplan–Meier plots for all four IOP criteria can be found in Figure 3.

### 3.2 Secondary endpoints

The mean number of topical IOP‐lowering medications was significantly lower at all timepoints compared with baseline and reduced from 3.4 ± 0.8 to 0.9 ± 0.9 at 12 months after surgery and 43% of patients were drop free (Tables 3 and 4). There was a significant reduction in medications between 3 months and 6 months (*p* = 0.0001) and 3 months and 12 months (*p* = 0.0021), consistent with the removal of the intraluminal Prolene stent between 3 and 6 months in 35 out of 49 patients. 23 (47%) cases were medication free after 12 months and 12 (24%), 12 (24%), 2 (4%) needed 1, 2 and 3 number of IOP‐lowering medications respectively (Table 3). Subgroup analysis revealed a significantly higher number of IOP‐lowering medications at 1 month kin the group with oil‐filled eyes (*p* = 0.0130) with no difference in IOP at any timepoint (Table S1).

**TABLE 3:** Use of intraocular pressure lowering medications before and after surgery.

	Preoperative	Week 1	Month 1	Month 3	Month 6	Month 12
Number of topical IOP‐lowering agents Mean ± SD	3.4 ± 0.8	0.5 ± 1.1	1.5 ± 0.9	1.6 ± 0.8	0.8 ± 0.9	0.9 ± 0.9
Number of eyes on topical IOP‐lowering agents (%)	49 (100)	9 (18)	39 (80)	41 (84)	23 (47)	26 (53)
Number of patients on systemic acetazolamide (%)	14 (29)	2 (4)	0	0	0	0

**TABLE 4:** Number of cases by number of glaucoma medications at 12‐month visit.

Number of IOP‐lowering agents	Cases (%)
0	21 (43)
1	12 (24)
2	12 (24)
3	2 (4)
4	0

No intraoperative complications occurred. A full overview of post‐operative complications during the 1‐year follow‐up period can be found in Table 5. BCVA was 0.50 ± 0.45logMAR before surgery and 0.52 ± 0.43logMAR at the 12‐month follow‐up visit. Loss of visual acuity occurred in five cases. Two cases were due to cataract progression, one case due to ocular ischaemic syndrome, one case due to hypotony with irregular astigmatism and one was secondary to corneal decompensation. Mean ECD pre‐ and 12 months post‐operative was 2244 ± 416 and 2178 ± 573 cells/mm^2^ respectively (*p* = 0.1374). Mean difference was −82.3 (95% CI −192:27.6), equivalent to 4.5 ± 15%. ECD loss >20%, >30% and > 40% occurred in four (8%), three (6%) and two (4%) cases. Corneal decompensation occurred in one case, which had a low endothelial cell count pre‐operatively (1127 cells/mm^2^).

**TABLE 5:** Complications of Paul glaucoma implant surgery.

	Early (<3 months) number of eyes (%)	Late (≥3 months) number of eyes (%)
General complications	5 (10)	7 (14)
Clinical hypotony	3 (6)	2 (4)
Choroidal detachment	1 (2)	2 (4)
Maculopathy	1 (2)	0
AC shallowing	1 (2)	2 (4)
Hyphaema	2 (4)	0
IOP spike	0	0
Corneal decompensation	0	1 (2)
BCVA loss	n/a	5 (10)
Macular oedema	0 (0)	0 (0)
Specific complications	6 (12)	1 (2)
Leak	0	0
Ptosis	0	0
Diplopia	0	1 (2)
Corneal dellen	0	0
Dysesthesia	0	0
Iridodialysis	0	0
Iris atrophy	0	0
Device obstruction	0	0
Device malposition	1 (2)	0
Device migration	0	0
Device exposure	0	0
Stent exposure	5 (10)	0

None of the six phakic cases underwent cataract surgery within 1 year after PGI implantation. Conjunctival erosion from the tip of the Prolene stent was managed by trimming the protruding tip in the slit lamp. Two days after the initial PGI implantation one case had the sulcus placed tube repositioned due to malposition.

Early and late clinical hypotony occurred in three and two cases respectively. Clinical hypotony was only found in eyes with previous glaucoma surgery. The first case of early hypotony was a child with Sturge–Weber that had AC shallowing, which resolved with cycloplegia. The second case had chronic uveitis and developed hypotony maculopathy which was unresponsive to cycloplegia and ophthalmic viscosurgical device (OVD, Healon GV Pro), (Johnson & Johnson, California, USA) in the AC, but resolved after temporary ligation with an absorbable suture (Vicryl 7–0, Ethicon, V546G). The third case did not resolve with OVD and the patient wanted a permanent ligation (Ethilon 8–0, Ethicon, B7714) of the tube. Both cases of late clinical hypotony occurred after intraluminal prolene stent removal at 14 weeks post‐operatively. Cycloplegia and OVD were followed by temporary ligation of the tube with an absorbable suture. Clinical hypotony resolved in one case whereas it reappeared in the other case. Stenting the tube with a Prolene 7–0 (Ethicon, W8702) resulted in resolution of choroidal effusions and a deep chamber but IOP did not increase above 6 mmHg and at the 1‐year visit refraction was unstable.

## 4 DISCUSSION

The current study presents the results from a consecutive case series after a complete transition in practice pattern from Baerveldt and Ahmed GDD to PGI. Key findings were that 71% of patients achieved an IOP under 12 mmHg and that complications were acceptable compared with other classical types of glaucoma surgery. Novel findings presented in this study include data on endothelial cell loss and differences in post‐operative course between oil‐filled eyes and other eyes.

### 4.1 Intraocular pressure

Previous reports on the IOP‐lowering efficacy of PGI report 12 months of pressure between 12.0 and 13.2 mmHg on a mean number of IOP‐lowering medications of 0.3 to 1.25 (José et al., 2022; Koh et al., 2020; Vallabh et al., 2022; Weber et al., 2023). Our result of 11.4 mmHg on 0.9 medications is thus comparable to previous reports. IOP success rates based on four different criteria (≤ 21, ≤18, ≤15, ≤12 mmHg) differed from those reported by Weber et al. where our complete success rates at ≤21 and ≤18 mmHg were lower. The complete success rates of 41% and 33% at ≤15 and ≤12 mmHg, respectively, were comparable to those reported by Weber et al. (46.7% and 22%). Qualified success rates were comparable at ≤21 and ≤18 whereas the qualified success rates at ≤15 and ≤12 may be a bit higher in the current study (86% and 71% vs. 62% and 27%). Two studies used a different success criterion (IOP <21 mmHg and >20% IOP reduction, with or without medications) as the primary outcome and with this definition of success, the data presented in this study (43% success without medication and 94% success with medications) are also comparable to the previous two studies (93.2% and 90.1% with medications and 38.4% and 68.9% without medication) (Koh et al., 2020; Vallabh et al., 2022). The profile of complete and qualified success rates at different IOP thresholds in this study is very similar to the ones reported by Vallabh et al. but differs from those reported by Koh et al. and Weber et al. In the current study, and probably also the one by Vallabh et al., IOP > 15 mmHg was rarely accepted because of glaucoma severity, leading to more liberal use of IOP‐lowering medications in these patients. Therefore, complete success rates are lower at the high IOP thresholds, but most importantly complete success rates are similar at the lower thresholds in all studies. A more aggressive approach with IOP‐lowering medication also explains the higher qualified success rates at the low IOP thresholds in our study and the one by Vallabh et al. compared to Webber and Koh et al. So, to summarise, the efficacy of the PGI appears similar in all studies but the lack of a common agreement on when to commence IOP‐lowering medications leads to different success rate profiles.

The study design and patient cohort in the study by Weber et al. are very similar to the current study. The major difference is that in the study by Weber et al. surgery was performed by a surgeon with vast experience with the PGI whereas in our study both surgeons were novices, and their learning curve is included in the data in this article. Our study thus demonstrates that PGI can be adopted in clinical practice without a learning curve delaying the results one can expect in the long run.

Subgroup analysis revealed that silicone oil‐filled eyes required more topical medications in the early post‐operative period to achieve pressure control. However, at 6 and 12 months, after stent removal, there was no difference in IOP or use of medications between oil‐filled eyes and the rest of the group. Earlier stent removal (before 3 months) may be an option in oil‐filled eyes, at least in cases with inadequate early pressure control on medications.

If the outcomes in this study are compared to Baerveldt and Ahmed tubes we find that the 1‐year IOP of 11.4 ± 3.3 mmHg reported in this study is at least comparable, or maybe favourable, to previous landmark trials (Ahmed Baerveldt Comparison study, Ahmed vs. Baerveldt study and Tube vs. Trabeculectomy trial) on Ahmed and Baerveldt tubes (Budenz et al., 2011; Christakis et al., 2011; Gedde et al., 2007). One‐year IOP for Baerveldt tube in these trials was 13.8 ± 4.8, 13.2 ± 6.8 and 12.4 ± 3.9 mmHg. Corresponding numbers for Ahmed tube were 15.4 ± 5.5 and 16.5 ± 5.3 mmHg. Two things are important to highlight when the Ahmed and Baerveldt data are compared with PGI in this study. The first is that the use of IOP‐lowering medications was not higher in this study, more the opposite. An average of 0.9 ± 0.9 medications were used in this study. The Baerveldt groups were on average 1.2 ± 1.3, 1.5 ± 1.4 and 1.3 ± 1.3 medications in the previous reported trials and the Ahmed groups were on average 1.8 ± 1.3 and 1.6 ± 1.3 medications respectively. The second thing to highlight is the higher standard deviation for both IOP and topical medications for both Ahmed and Baerveldt compared to PGI in this study. This suggests that PGI provides a more predictable outcome, which facilitates patient information prior to surgery.

### 4.2 Complications

The theoretical advantage of a tube with a smaller diameter is a reduced risk for tube erosions and better chances of positioning the tube as far away as possible from the cornea in the chamber angle. No tube erosions occurred in this study. We did, however, note one corneal decompensation and two eyes experiencing a more than 40% decrease in ECD count. It is important to highlight that corneal decompensation was only observed in a cornea with a low ECD count before surgery (1127 cells/mm^2^). For this reason, it would probably have been wiser to place the tube in the sulcus instead of the AC. The average ECD change for the whole group was −4.5% and comparable with the numbers reported by Panarelli et al. 1 year after trabeculectomy or PreserFlo Microshunt (6.9% and 5.2% respectively) (Panarelli et al., 2024). In the short term, PGI implantation does not appear to have a more detrimental effect on the cornea than other types of glaucoma filtration surgery, with or without a device in the AC. The short follow‐up period of 12 months does not allow us to fully appreciate if the smaller tube provides any benefit compared with Ahmed and Baerveldt in terms of tube erosion risk and ECD loss and a longer follow‐up is needed to answer this question. Other reports on ECD after PGI implantation have not been published. Whereas it appears worrying that four eyes lost >2 lines of BCVA it is important to note that many of the eyes in this study were frail and with co‐morbidities including neovascular glaucoma secondary to ocular ischaemic syndrome, advanced glaucoma in oil‐filled eyes, chronic uveitis, etc., and other causes of the loss of visual acuity than the surgery per se is likely. In addition to this cataract progression was the cause in two cases.

Five cases (10%) developed clinical hypotony, of these two after stent removal. Four cases needed intervention and were initially treated with OVD injections in the AC but were eventually treated with ligation of the tube. The use of OVD injections is comparable to the use reported by Koh et al. (9.5%) and Tan et al. (8.9%), but markedly different from the study by Vallabh et al. and Weber et al., who reported OVD injection in none and one eye (1.8%) respectively. Ligation of the tube was successful in treating hypotony. Intraluminal stent was removed more often and earlier than both Vallabh et al. and Weber et al. and a more conservative approach would have reduced the hypotony rate in this study. Even so, hypotony rates reported in this study and the other PGI studies appear lower compared to three landmark trials on Baerveldt and Ahmed tubes. The TVT study (Baerveldt) reported choroidal effusions in 16% of eyes and flat/shallow ACs in 11% of eyes. The ABC and AVB study (Baerveldt vs. Ahmed) reported shallow anterior chambers in 14–20% of eyes and choroidal effusions in 10–15% of eyes.

### 4.3 Study population, design and reporting

Although the study is not registered as prospective trial the quality of the data in this study is comparable to that of a prospective trial (Abegao Pinto et al., 2023). The clinical course of the patients was pre‐defined and standardised and all data were collected according to pre‐defined criteria. Parameters on efficacy and safety were chosen in accordance with the EGS guideline on surgical innovation to facilitate transparent reporting and comparison with other studies. The patient cohort represents all tube surgeries at our centre during the time period except one (Ahmed GDD, requested by the patient). The lost to follow‐up rate in this study was minimal (4%) and could not have been reduced (one death and one open globe trauma with loss of light perception). The study design, where PGI was introduced as the sole GDD from start and data collected from all cases, means our results are not subject to case selection or selection bias and provides an indication of the results that can be achieved in a mixed group of patients. It also means that a possible learning curve is included, which may have a negative impact on the results and thus may underestimate the device. The patient cohort in this study reflects the clinical practice at our centre, where GDD is not first‐line glaucoma surgery but reserved for patients where the risk for failure after trabeculectomy is considered increased. Thus, all eyes included in this study had risk factors for failure, for example, previous filtration surgery, neovascular glaucoma, uveitis or oil‐filled eyes. At centres that use GDD as first choice procedure for filtration surgery one can speculate if success rates would be higher than that reported in our study. It is also worth noticing that the vast majority of eyes were Caucasian. This represents the demographics in Denmark. The observational nature of this study with only one treatment arm does not permit a conclusion of whether PGI has a better IOP‐lowering effect than Ahmed and Baerveldt. The results presented in this paper are, however, at least comparable to the results presented in the large randomised trials of Ahmed and Baerveldt.

We conclude that it was possible to change practice from using two different GDDs (Ahmed and Baerveldt) to only PGI in a widespread spectrum of glaucoma patients in need of a GDD. The IOP‐lowering effect was comparable to published data on Baerveldt tubes but with a predictable IOP reduction from postoperatively day one and an acceptable complication rate. Patients in need of GDD are often complex and may need IOP in the low teens to prevent further vision loss. Almost three out of four patients in this study achieved an IOP below 12, which for most patients will be a satisfactory outcome. The current study adds to the increasing number of retrospective studies presenting good outcomes with PGI and adds new knowledge in terms of early endothelial cell loss. Randomised clinical trials comparing PGI with, for example, Ahmed and Baerveldt or other GDDs are needed to fully understand the efficiency and safety of PGI compared with gold standard GDDs.

## Supporting information


Data S1.
