# Supplementary material for: One tube for all: 1‐year outcomes after transition to Paul glaucoma implant at a tertiary centre
Source: Acta Ophthalmol. 2025 Jan 24;103(4):461–8. doi: 10.1111/aos.17443 (PMC12069969; doi:10.1111/aos.17443)
Supplement: Supplementary file 1 — Data S1. [file AOS-103-461-s001.docx]

| Supplementary Table 1. Intraocular pressure and intraocular pressure lowering medications after Paul Glaucoma Implant surgery. | | | | | | | |
| --- | --- | --- | --- | --- | --- | --- | --- |
|  | Baseline | 1 day | 1 week | 1 month | 3 month | 6 month | 12 month |
| IOP  mmHg, mean ± SD | | | | | | | |
| All eyes (49) | 29.9 ± 8.6 | 17.7 ± 8.5 | 16.6 ± 7.5 | 13.7 ± 5.5 | 14.2 ± 5.6 | 12.6 ± 3.7 | 11.4 ± 3.1 |
| Oil filled eyes (13) | 3.3 ± 7.7 | 16.7 ± 4.1 | 22.2 ± 8.7 | 16.8 ± 7.3 | 14.8 ± 5.6 | 13.7 ± 4.8 | 12.2 ± 3.7 |
| PGI in sulcus (7) | 30.0 ± 8.0 | 25.7 ± 10.0 | 17.6 ± 10.3 | 15.3 ± 4.9 | 15.0 ± 4.5 | 11.9 ± 2.9 | 11.9 ± 2.0 |
| Non oil filled eyes with PGI in the AC (28) | 28.8 ± 9.3 | 15.5 ± 8.1 | 13.7 ± 4.4 | 12.2 ± 3.8 | 13.7 ± 6.1 | 12.3 ± 3.4 | 10.9 ± 3.0 |
| Medication  mean number of topical medications ± SD  % patients treated with acetazolamide | | | | | | | |
| All eyes (49) | 3.4 ± 0.8  29 | 0 | 0.5 ± 1.1  4 | 1.5 ± 0.9 | 1.6 ± 0.8 | 0.8 ± 0.9 | 0.9 ± 0.9 |
| Oil filled eyes (13) | 3.4 ± 0.8  23.1 | 0 | 0 | 2.0 ± 0 | 1.9 ± 0.6 | 0.9 ± 0.9 | 1.2 ± 0.8 |
| PGI in sulcus (7) | 3.4  71.4 | 0 | 1.4  14.3 | 1.7 | 0.9 | 0.6 | 0.7 |
| Non oil filled eyes with PGI in the AC (28) | 3.3 ± 0.8  20 | 0 | 0.3 ± 0.8 | 1.3 ± 0.9 | 1.8 ± 0.7 | 0.8 ± 0.7 | 0.8 ± 0.9 |

Abbreviations: IOP, intraocular pressure; SD, standard deviation; PGI, Paul Glaucoma Implant; AC, anterior chamber.
